# Proficiency of Nucleic Acid Tests for Avian Influenza Viruses, Australasia

**DOI:** 10.3201/eid1407.071098

**Published:** 2008-07

**Authors:** Sacha Stelzer-Braid, Ros Escott, Cristina Baleriola, Peter Kirkland, Peter Robertson, Michael Catton, William D. Rawlinson

**Affiliations:** * Prince of Wales Hospital, Randwick, New South Wales, Australia; † University of New South Wales, Kensington, New South Wales, Australia; ‡ Royal College of Pathologists of Australasia, North Ryde, New South Wales, Australia; § Elizabeth Macarthur Agricultural Institute, Camden, New South Wales, Australia; ¶ Victorian Infectious Diseases Reference Laboratory, North Melbourne, Victoria, Australia

**Keywords:** Avian influenza, pandemic, quality control, reverse transcriptase polymerase chain reaction, dispatch

## Abstract

An avian influenza quality assurance program was used to provide information for laboratories on the sensitivity and specificity of their avian influenza nucleic acid testing. Most laboratories were able to correctly detect clinically relevant amounts of influenza virus (H5N1), and results improved as each subsequent panel was tested.

Highly pathogenic avian influenza (HPAI) virus (H5N1) is endemic among the world’s wild bird populations and continued to spread during 2006 to poultry across Asia, Africa, and mainland Europe (*1*,*2*). Sensitive, specific diagnostic methods are essential for early accurate detection of HPAI virus in the prepandemic and early pandemic phases in countries where no cases have been recorded, such as Australia (*3*).

Several sublineages of HPAI (H5N1) exist (*4*,*5*). Virus mutation requires that nucleic acid testing (NAT) methods such as reverse transcription–PCR (RT-PCR) be continually improved to remain sensitive for emerging strains (*6*–*12*). Currently, the World Health Organization (WHO) recommends an RT-PCR based on primers published in 1998 (www.who.int/csr/disease/avian_influenza/guidelines/RecAIlabtestsAug07.pdf).

## The Study

We report results from an avian influenza quality assurance program (QAP) that used an established, Internet-based quality assurance reporting system (www.rcpaqap.com.au/serology), allowing remote data entry, rapid result dissemination, and expert comment. The QAP provided feedback to laboratories on NAT characteristics (PCR accuracy, sensitivity, and specificity), reporting optimization, and assessment of continuously updated laboratory-developed NAT methods.

During 2006, three panels of specimens were distributed to 29 participating laboratories: 15 from Australia (including 4 veterinary laboratories); 2 from Hong Kong Special Administrative Region, People’s Republic of China; 5 from Singapore; 1 from New Caledonia; 1 from Malaysia; and 5 from New Zealand. The panels consisted of an Indonesian and a Vietnamese strain of avian influenza virus (H5N1), originally isolated from humans and grown in MDCK cells. Viral copy numbers were estimated by comparing real-time RT-PCR crossing-point values to a standard curve generated by using plasmid standards; the amplicon was cloned into pGEMT-Easy (Promega, Madison, WI, USA). Plasmid standard concentrations were estimated as described previously (*13*) and as recommended by the LightCycler manufacturer (Roche, Indianapolis, IN, USA). Sensitivity of NATs was determined with a range of clinically relevant nucleic acid concentrations of both influenza (H5N1) strains (10^3^ to 10^–1^ copies/μL) to enable laboratories to assess limit of detection (LOD) of their assays. Specificity was assessed by inclusion of other influenza strains and a negative control (Table 1). All strains and MDCK cells were inactivated by exposure to 50 KGy of γ-irradiation, except for strain A (H7N4), which was inactivated by the addition of lysis buffer (*14*).

**Table 1:** Avian influenza quality assurance panel specimen details, Australasia

Specimen	Dilution	Copy number/μL
Influenza A (H5N1) Indonesian	1:1,000	6.25 × 10^3^
Influenza A (H5N1) Vietnamese	1:1,000	5 × 10^3^
Influenza A (H5N1) Indonesian	1:100,000	6.25 × 10^1^
MDCK-negative control	1:1,000	NA
Influenza A (H5N1) Vietnamese	1:100,000	5 × 10^1^
Influenza A (H3N2) (isolated in Sydney, New South Wales, Australia; close sequence match to A/Canterbury/29/2005)	1:1,000	1.25 × 10^2^
Influenza B (isolated in Sydney; no sequence information available)	1:1,000	2 × 10^2^
Influenza A (H7N4) (A/emu/NSW/97)	1:1,000	1 × 10^4^
Influenza A (H5N1) Indonesian*	1:10,000,000	6.25 ×10^–1^
Influenza A (H5N1) Vietnamese*	1:10,000,000†	5 × 10^–1^

*These dilutions were included in panels 2 and 3 only; NA, not applicable. †For panel 3, the dilution for the influenza (H5N1) Vietnamese strain was changed to 1:1,000,000, with a copy number of 5 × 10^0^ μL.

Four experiments to define optimum conditions were conducted. 1) LOD determinations, with a dilution series of all strains, were tested by using real-time RT-PCR (*15*). 2) Transport media were compared by using serial dilutions of inactivated influenza virus (H5N1) in phosphate-buffered saline with gelatin (with antimicrobial agents) (PBSG), TE buffer, and buffer RLT (lysis) (QIAGEN, Valencia, CA, USA), placed at –80°C, –20°C, +4°C, +25°C, and +37°C for 10 days. Each day, 1 tube at each temperature condition was removed, and viral DNA was extracted by using the QIAamp viral RNA minikit (QIAGEN) and tested with a real-time RT-PCR (*15*). 3) For further stability testing, a test panel diluted in PBSG was sent by courier from Sydney to Hong Kong, held by Australian customs for 7 days, and returned unopened 15 days after dispatch. The temperature range the shipped panel was exposed to is unknown; however, previous temperature loggers have recorded temperatures from 22°C to 33°C. The panel that traveled was tested against a panel that had been stored optimally (–80°C) (*15*). No difference was detected in the amount of virus in the specimens that traveled compared with optimally stored specimens, indicating that the specimens were stable under normal transport conditions (results not shown). 4) Homogeneity was established before distribution by having the panel tested and approved by 2 reference laboratories (Victorian Infectious Diseases Laboratory, Melbourne, Victoria, Australia; Western Australian Centre for Pathology and Medical Research, Perth, Western Australia, Australia) and 1 animal reference laboratory (Elizabeth Macarthur Agricultural Institute, Camden, New South Wales, Australia).

For each panel the samples were diluted in PBSG and transported by courier at ambient temperature in 3 seasons (autumn, winter, summer). Participants were not required to use a certain NAT method. Participants were asked for information on methods used, including extraction and RT-PCR protocol and primer/probe sequences. A total of 780 results were analyzed, and a report was issued to participants within 3 weeks of each survey closing, well before the next panel shipment. This allowed participants to adjust their testing procedures if necessary before the next survey began. Results were reported by participants as positive, negative, or equivocal. For simplicity, we report equivocal results as positive, given that participating laboratories retest an equivocal result and generally do not report such results as negative. On average for the 3 panels, 2.6% of results were reported as equivocal.

Panel 1 contained 8 specimens, which included 2 dilutions (10^3^ and 10^1^ copies/μL) of each subtype H5N1 strain (Table 1). Only 35% of participants correctly identified all samples; 95% reported a correct result for the highest concentration of both subtype H5N1 strains (10^3^ copies/μL); 70% could detect the lower concentration (10^1^ copies/μL). Only 46% of participating laboratories used an influenza A matrix assay as well as a specific H5 assay (Table 2). Laboratories were advised to use both methods in tandem to reduce the chance of missing variant influenza (H5N1) strains that might not be detected by their specific H5 assay. Some false positives (1.3%) were reported, and some confusion regarding terminology occurred: many laboratories reported results as for subtype H5N1 assays, despite most of these results being specific for the H5 gene only.

**Table 2:** Summary of avian influenza quality assurance project, Australasia*

Panel no.†	Date	% Correct results, Indonesian strain‡	% Correct results, Vietnamese strain‡	% Testing for influenza A matrix	Most common extraction method (% participants)	Most common amplification method (% participants)
1	2006 Oct 7	87.5	80	46	QIAGEN QIAamp viral RNA minikit (50)	QIAGEN Artus Influenza/H5 LC RT-PCR kit (20)
2	2006 Nov 9	84	84	73	QIAGEN QIAamp viral RNA minikit (50)	Invitrogen Superscript III qRT-PCR (31)
3	2006 Jun 11	82	88	75	QIAGEN QIAamp viral RNA minikit (50)	Invitrogen Superscript III qRT-PCR (40)

*RT-PCR, reverse transcription–PCR. †Panels 2 and 3 had 2 very low dilutions of subtype H5N1 that were beyond the limit of detection for most laboratories. Percentage of correct results reported for the Indonesian and Vietnamese strains, with these results included, is 59 and 59 for panel 2 and 56 and 66 for panel 3, respectively. ‡Results reported for influenza H5/(H5N1) testing.

For panel 2, no participants correctly identified all samples because of the addition of 2 extremely dilute samples of influenza virus (H5N1) (10^0^ and 10^–1^ copies/μL) that were below the LOD for most laboratories. Eleven percent of participants detected 1 strain of HPAI virus (H5N1) by using primers specific for H5 or subtype H5N1 at 1 of the 2 highest dilutions, but not both. In our experience, dilute specimens are useful for assessing the LOD of the testing system because they may highlight the most sensitive methods available. The number of laboratories using a generic influenza A test, in addition to a specific H5 test, increased to 73% (Table 2).

For panel 3, sensitivity of detection improved compared with panel 2: 25% of participants detected a strain of influenza virus (H5N1) at the lowest concentrations. Sensitivity of H5/H5N1 testing for the influenza (H5N1) Vietnamese strain increased over time, while sensitivity of testing decreased slightly over the 3 panels for the influenza (H5N1) Indonesian strain (Table 2). Laboratories had altered primer/probe sets to increase sensitivity for the Vietnamese strain, which resulted in decreased sensitivity for the Indonesian strain. Sensitivities of other testing methods (influenza A, B, H3) increased during subsequent testing of each panel (data not shown); the number of correct results reported by participants using influenza A matrix testing rose from 84% in panel 1 to 91% in panel 3.

## Conclusions

Most participants did not disclose their primer/probe sequence information, which made it difficult to recommend the most sensitive methods to other participants. However, during a prepandemic phase, having a range of primers/probes being used may be optimal, providing influenza A matrix detection is also conducted and QA is maintained, until WHO recommends a method to detect new pandemic strains.

Participants in the avian influenza QAP made clear improvements in the sensitivity and specificity of their NAT methods over time. It is important to provide continuing QA to expose inconsistencies in results or primers that may be skewed toward a particular strain.
